# Advances in research on dental occlusion and brain function: neuroimaging correlates and molecular mechanisms

**DOI:** 10.3389/fneur.2026.1841493

**Published:** 2026-09-07

**Authors:** Bingfeng Dai, Ziming Zhao, Jie Lin, Xiaochuan Yang, Fanghong Yang, Zhina Wu

**Affiliations:** 1 School of Stomatology, Shandong Second Medical University, Weifang, Shandong, China; 2 Department of Orthodontics, Weifang People’s Hospital, Shandong Second Medical University, Weifang, Shandong, China

**Keywords:** brain function, brain structure, correlation, neuroimaging, occlusion

## Abstract

The relationship between dental occlusion and brain function is bidirectional, involving neuroanatomical, neurophysiological, and clinical disorders. Occlusal sensory input modulates sensorimotor control, cognitive functions, and emotional states via neural pathways, while aberrant brain function can conversely disrupt occlusion homeostasis. This review synthesizes recent progress in deciphering the bidirectional interactions between dental occlusion and brain function, with a focus on the impacts of occlusion on brain structure and function, the underpinning molecular and physiological mechanisms, as well as the neural regulatory pathways governing occlusion. Crucially, this synthesis evaluates evidence strength, distinguishes animal models from human clinical studies, accounts for confounding factors such as periodontitis and systemic inflammation, and highlights methodological limitations in current neuroimaging research. These findings hold substantial implications for basic neuroscience research, clinical dentistry, and the interdisciplinary management of dental and neurological comorbidities.

## 1 Background

Occlusion refers to the contact relationship between the upper and lower teeth, including the state in which the teeth are not in direct contact during mastication, as well as the concept of dental occlusion (1). For clarity, key concepts should be distinguished: malocclusion denotes structural deviations from ideal anatomical alignment; occlusal disturbance/interference refers to transient or acute disruptions in tooth contact; and occlusal dysfunction represents functional impairment, including tooth loss and loss of occlusal support (2). As the core function of the chewing system, occlusion is an important part of oral function, playing a key role in activities like chewing, swallowing, and speaking. The information from occlusion is sent to the brain through sensory organs such as the mechanoreceptors in the periodontal ligament and proprioceptors in the chewing muscles, which modulate the activity of brainstem nuclei. This, in turn, regulates the movement of the muscles in the mouth and jaw (3). The brain, as the central part of the nervous system, controls body movements, including those of the mouth, by using nerve fibers, brain-derived neurotrophic factor (BDNF), and neurotransmitter (4, 5). Studies show that the connection between dental occlusion and brain function is not only seen in the brain’s structure but also in research on brain imaging and behavior, which link occlusion to cognitive function, emotions, and certain health problems (6). Understanding the relationship between dental occlusion and brain function is important for learning how the oral and nervous systems interact, and for preventing and treating related conditions.

## 2 Association between dental occlusion and specific brain regions

### 2.1 Brain regions associated with occlusion

Dental occlusion triggers a sequential neural pathway spanning peripheral receptors, trigeminal nucleus, thalamus, and cortex, forming a distributed activation pattern that integrates sensation, motor control, interoception, and cognition. Mandibular movements and occlusal actions dynamically engage multiple brain regions, key among which are:

#### 2.1.1 Sensorimotor cortex

The primary somatosensory cortex (SI) and primary motor cortex (MI) function as the core hubs for occlusal signal processing. The orofacial somatotopic map within SI decodes fine-grained sensory inputs, such as occlusal force magnitude and contact position. Meanwhile MI regulates masticatory muscle contraction via corticobulbar projections, thereby orchestrating the execution and real-time refinement of occlusal movements (7, 8). Elevated occlusal force elicits a linear increase in blood oxygen level dependent (BOLD) signals in SI, MI, and the anterior cerebellum, reflecting a direct correlation with bite strength perception and motor control precision (9).

#### 2.1.2 Supplementary motor area and prefrontal cortex

The supplementary motor area (SMA) contributes to rhythmic modulation and motor planning of occlusal movements, whereas the dorsolateral prefrontal cortex (PFC) integrates cognitive aspects of occlusion, such as adjusting bite strategies according to food texture and coordinating the timing of mastication and swallowing (7, 10).

#### 2.1.3 Cerebellum and basal ganglia

The anterior cerebellum and vermis are engaged in the coordination of occlusal movements, continuously correcting motor errors in real time. The basal ganglia including the caudate nucleus, contribute to the learning and habituation of occlusal actions via dopaminergic pathways (7, 9).

#### 2.1.4 Insula and anterior cingulate cortex

The insula integrates visceral sensory and emotional signals related to occlusion, while the anterior cingulate cortex (ACC) is involved in the emotional processing of occlusal pain and discomfort. Together, these regions mediate the somatosensory–emotional interactions triggered by occlusal disturbances (11, 12).

### 2.2 Comparison of neuroimaging modalities and structural plasticity

Neuroimaging techniques provide critical insights into occlusion-related central activity; however, each modality exhibits distinct technical strengths and limitations (Table 1).

**Table 1:** Critical comparison of neuroimaging modalities in dental occlusion research.

Modality	Spatial resolution	Temporal resolution	Primary application in occlusion research	Strengths	Limitations
**fMRI**	High (~1–3 mm)	Low (~1–2 s)	Task-based cortical/subcortical activation & resting-state network connectivity (7, 9, 11)	Excellent deep-structure resolution	Motion artifact sensitivity during active chewing
**fNIRS**	Moderate (~10–20 mm)	High (~10–100 ms)	Cortical hemodynamics during upright chewing or denture wearing (15, 53)	Motion-tolerant, allows natural upright mastication	Limited to superficial cortex; lower spatial resolution
**EEG**	Low (~cm)	Very High (~ms)	Neurophysiological dynamics and cortical spectral power (59, 60)	High temporal tracking of cortical excitability	Low spatial resolution; vulnerable to muscle artifacts
**PET**	Moderate (~4–5 mm)	Very Low (minutes)	Metabolic rate and receptor binding studies (6, 51)	Direct quantification of neurochemical systems	Ionizing radiation; low temporal resolution
**DTI**	High (~1–2 mm)	N/A	White matter tract integrity mapping (19, 51)	Resolves structural fiber connectivity	Complex fiber-crossing resolution

fMRI, functional magnetic resonance imaging; fNIRS, functional near‑infrared spectroscopy; EEG, electroencephalography; PET, positron emission tomography; DTI, diffusion tensor imaging.

Occlusal disturbances or pathological malocclusion are associated with alterations in cerebral blood flow and brain structure. Experimental occlusal disturbances lead to increased BOLD signals in the contralateral SI, accompanied by coordinated activation of MI, which regulates the contraction strength and rhythm of masticatory motor units to compensate for aberrant occlusal contacts. Clenching activates the SI and MI brain regions, and this pattern is highly repeatable across different imaging methods. However, early fMRI and EEG studies relied on small test groups. Therefore, researchers need to confirm these results in larger and standard groups. This is further associated with bilateral activation and short-term plasticity changes in the SMA, thalamus, insula, cerebellum, and PFC, with cortical activation returning to baseline approximately 60 min after removal of the disturbance (11, 13, 14). Functional near-infrared spectroscopy (fNIRS) demonstrates that mandibular retrusion malocclusion in healthy individuals elicits significant activation of the PFC, which positively correlates with subjective discomfort as measured by the visual analog scale (15). In patients with skeletal open bite malocclusion, changes in cerebral blood flow during mastication are influenced by the number of occlusal contact points and mandibular movement parameters, with a reduced magnitude of blood flow increase compared to healthy controls (16). Patients with mandibular prognathism malocclusion exhibit a diminished increase in blood flow within the inferior frontal gyrus during mastication (17). Unilateral mastication may result in lateralized cerebral activation, potentially influenced by hand dominance and habitual chewing side; however, the functional asymmetry between hemispheres remains a topic requiring further investigation (13, 14).

Tooth loss, a common occlusal dysfunction, can also impact cerebral blood flow and brain structure. During normal mastication, increased blood flow is observed in the sensorimotor cortex, PFC, and other related brain regions (18). Human observational studies show that individuals with tooth loss exhibit reductions in total brain volume and gray matter (19). These reductions occur particularly in the paracentral lobule, precuneus (20), and cognition-related regions like the hippocampus and caudate nucleus (21). Furthermore, this cerebral atrophy correlates with cognitive decline (22, 23). Animal studies indicate that tooth loss results in reduced gray matter in the primary sensorimotor cortex, increased volume in brainstem sensorimotor nuclei and limbic structures, such as amygdala, with genetic background modulating regional structural alterations (24). However, cross-sectional imaging studies cannot determine the temporal sequence of cerebral structural and functional changes relative to occlusal alterations. Therefore, future longitudinal follow-up studies are warranted to elucidate the precise temporal trajectory of cerebral structural and functional alterations associated with the onset, progression, and post-therapeutic intervention of occlusal abnormalities. As a core oral function, occlusion can influence cerebral blood flow and structural integrity through multiple neural reflex pathways, thereby impacting oxygen and energy supply to brain tissue and key functions such as cognition. Investigating these relationships is crucial for understanding the interplay between oral health and central nervous system (CNS) function and has significant theoretical and clinical implications for promoting brain health.

### 2.3 Modulation of memory and cognitive function by occlusion

Memory is the psychological process of encoding, storing, and retrieving information, while cognition refers to the brain’s capacity to represent and process external reality. Memory constitutes a critical component of cognitive function, influencing daily life and behavior (25). Occlusal status is closely associated with memory and cognitive performance (26–28).

#### 2.3.1 Rodent models and translational considerations

Rodent models indicate that molar extraction leads to structural alterations in sensorimotor, cognitive, and limbic systems (24), including decreased pyramidal cell density and atrophy in the hippocampus, reduced dendritic spine number, and hypertrophy and proliferation of astrocytes (29–31), corresponding to impaired performance in spatial memory tasks. Occlusal dysfunction induced by tooth loss also alters hippocampal BDNF levels, decreases expression of synaptic plasticity-related genes and transmembrane receptor proteins (32–35), and reduces nitric oxide and its synthase levels (36), thereby impairing neurotransmitter release, synapse formation, molecular signaling, and ultimately cognitive and spatial learning abilities. Following restoration of missing teeth, hippocampal neuron density in mice recovers, accompanied by significant improvements in memory encoding and retrieval (37). Soft-diet feeding similarly reduces cortical synaptogenesis and hippocampal pyramidal cell density in mice (29, 32, 38), impairing spatial learning, suggesting that diminished masticatory activity may represent a risk factor for cognitive decline. However, when translating rodent data to humans, translational validity must be acknowledged: acute molar extraction in rodents induces significant surgical stress and immediate dietary shock, whereas human tooth loss is typically gradual and age-related. Despite these etiological differences, rodent models serve as a valid and necessary analog for isolating the neurobiological impact of reduced trigeminal sensory input, providing a relevant link to the cognitive decline observed in human clinical studies.

#### 2.3.2 Human observational evidence and confounding variables

A growing body of evidence supports a dose–response association between tooth loss and the risk of cognitive impairment (39–42). Epidemiological data demonstrate a linear relationship where the risk of cognitive decline and dementia increases progressively with the number of teeth lost (43). This risk is particularly pronounced when occlusal support is compromised; for instance, posterior tooth loss alone can elevate dementia risk by 2 to 3 times (19). Meta-analyses of observational cohorts report that tooth loss is associated with a moderate increase in dementia risk (44). In addition, attributing this cognitive risk solely to the loss of trigeminal sensory afferents overlooks critical confounding factors, as tooth loss is frequently a consequence of severe periodontitis—a condition where systemic inflammation and shared socio-behavioral variables independently influence cognitive trajectories (45). Furthermore, shared confounding variables—such as lower socioeconomic status, smoking, diabetes, and dietary deficiencies—substantially influence both dental loss and cognitive trajectories (45–47).

Conversely, cognitive function can influence occlusal behavior; patients with cognitive decline may exhibit masticatory dysfunction and impaired bite force control (48), likely related to disrupted coordination of masticatory muscles (49). Dementia patients frequently forget masticatory sequences or fail to adjust occlusal force according to food texture, resulting in reduced chewing efficiency and an increased risk of aspiration (50). Tooth loss in elderly individuals with cognitive impairment may be associated with medial temporal structural differences and cerebral nutritional supply (51). It can lead to parahippocampal atrophy and increased white matter hyperintensities. Both of these brain changes are linked to dementia (46). Altered masticatory function accompanying tooth loss interacts with cognitive function, potentially creating a “vicious cycle.”

Prosthetic rehabilitation plays a critical role in preventing cognitive impairment and serves as a protective factor for cognitive function in patients with dementia and neurodegenerative disorders (52). In edentulous elderly patients, denture use enhances PFC activation as measured by fNIRS, reducing differences relative to young healthy individuals, suggesting that prosthetic restoration can maintain prefrontal cognitive function by restoring occlusal sensory feedback (40, 53). Edentulous patients with mild cognitive impairment show near-baseline cognitive performance within 6 weeks after progressive implant-supported oral rehabilitation, with implant-supported prostheses demonstrating superior outcomes compared to conventional removable dentures (54). Collectively, these studies systematically demonstrate the close relationship between oral function and brain function, providing critical scientific evidence and practical guidance for clinical design of oral rehabilitation from a neuroregulatory perspective.

### 2.4 Effects of occlusal rehabilitation on brain plasticity

Brain plasticity refers to the brain’s capacity to adapt its structure and function in response to environmental stimuli and experience (55). Alterations in occlusal status can induce neuroplastic changes in the cerebral cortex. In rodents, molar loss leads to changes in neurotransmitter levels, including dopamine and acetylcholine; dietary modifications can promote the expression of BDNF in the contralateral hemisphere, activate hippocampal cell proliferation, and partially reverse neural damage (56). Restoration of masticatory stimulation can reverse hippocampal neuronal loss, decreased synaptic density, and spatial memory impairments caused by molar extraction (57). Denture rehabilitation in molar-deficient mice restores neuronal density in specific hippocampal regions, suggesting that occlusal support facilitates neural repair (37, 58).

In edentulous patients, denture use increases EEG α-wave power spectral density, reflecting a stabilization of brain functional state (59, 60). Implant-supported prostheses elicit stronger activation in memory-related brain regions, and the closer the prosthetic function approximates natural dentition, the more accurately the CNS sensory-motor feedback is restored (61, 62). These findings indicate that implant-supported complete dentures may better restore sensory and motor feedback, including tactile perception, stereognosis, and masticatory performance, compared with conventional prosthetic approaches.

Orthodontic treatment can also induce short-term neuroplastic changes in the CNS. Transcranial magnetic stimulation studies reveal enhanced cortical motor excitability following orthodontic intervention; after 30 days, these measures gradually return to baseline, demonstrating dynamic adaptation of the brain to occlusal alterations induced by orthodontics (63). Age significantly affects activation patterns; compared with younger individuals, older adults often exhibit bilateral compensatory activation, indicating an adaptive mechanism of neural plasticity in the aging brain. This plasticity is dynamic and partially reversible, although its long-term effects require further longitudinal investigation (7).

Overall, brain plasticity enables alterations in occlusal state to induce dynamic adaptations in brain structure and function through pathways such as neurotransmitter regulation and neurotrophic factor expression. Rehabilitation of occlusion through prosthodontic or orthodontic interventions can partially reverse neural damage, stabilize brain functional states, and provide core targets for clinical interventions linking oral health and brain function. Neuroplasticity thus holds promise as a novel indicator for assessing brain function, complementing clinical symptoms and neuroimaging evaluations.

## 3 Association between occlusion and clinical disorders

While occlusion-related clinical comorbidities, such as cardiac dysregulation, tinnitus, frailty, span multiple systemic domains, neuroimaging serves as the essential diagnostic and mechanistic bridge mapping these peripheral dysfunctions to central neural circuit realignments.

### 3.1 Occlusion and psychosocial factors

#### 3.1.1 Anxiety and depression

Depression and anxiety disorders are common psychiatric conditions, with abnormalities in the prefrontal-limbic circuitry and serotonergic projections considered shared neural bases for their comorbidity (64). Rodent models have demonstrated that occlusal disturbance may represent a risk factor for an organism’s hypersensitivity to novel stressors, suggesting an association between occlusion and the organismal stress response (65). Specifically, occlusal dysfunction is associated with heightened anxiety through both peripheral and central pathways involving the hypothalamic–pituitary–adrenal (HPA) axis and the 5-Hydroxytryptamine (5-HT) system, exacerbating anxious behavior (66)^.^ In humans, clinical observations demonstrate that chewing gum can alleviate anxiety and depressive symptoms (67). Current evidence suggests a potential bidirectional association between dental occlusion and the development of temporomandibular disorders (TMD), although causality in observational cohorts remains to be conclusively established (68). TMD patients frequently exhibit heightened anxiety, which is further aggravated by occlusal disturbance; treatment reduces both pain and anxiety, with decreases in anxiety corresponding to reduced insular activation on functional magnetic resonance imaging (fMRI) (12, 69). Thus, maintaining normal occlusal function or restoring appropriate masticatory stimulation may represent a potential intervention to modulate emotion-related neural circuits and improve anxiety and depression.

#### 3.1.2 Stress and strain

Stress is an emotional state triggered by dangerous or unexpected environmental changes, potentially influencing decision-making and psychological processes. Brain regions most affected by stress include the hippocampus, amygdala, and their connected PFC (70). Occlusal activity modulates stress responses; in rodent models, occlusal disturbance influences corticosterone secretion and 5-HT/5-HT₂ₐ receptor expression in the PFC, hippocampal CA1, and dentate gyrus, affecting emotional regulation (66). Additionally, via the connection between the trigeminal nerve and the locus coeruleus (71), occlusal dysfunction is linked to overactivation of the sympathetic-adrenal medullary system and the HPA axis, thereby influencing serum corticosteroid levels and contributing to chronic stress (72). In human clinical observations, appropriate mastication reduces stress-related endocrine responses, decreases hormone release, and alters PFC activity, ultimately attenuating stress and strain (73–76). Chewing, e.g., gum, is a simple means to relieve stress, though its precise mechanisms and individual susceptibility require further investigation.

### 3.2 Masticatory function and dementia

Alzheimer’s disease (AD) is a progressive neurodegenerative disorder (77). Occlusal dysfunction can influence the nervous system and contribute to AD pathogenesis (78). Numerous studies indicate that individuals with significant tooth loss have an increased risk of dementia (41, 79), with a linear relationship between tooth loss and cognitive decline: the more teeth lost, the higher the risk of cognitive impairment and dementia (43). Posterior tooth loss and loss of posterior occlusal support may increase dementia risk by 2–3 times (19); nevertheless, current clinical literature remains largely restricted to cross-sectional association, lacking prospective randomized controlled trials (RCT) evidence to establish whether occlusal dysfunction directly drives neurodegeneration. Furthermore, as previously discussed, these numerical risk estimates must be interpreted cautiously due to inherent methodological limitations, including reverse causation and shared clinical confounders. This association is likely due to greater reduction in sensory input to the brain, accelerating atrophy in hippocampal and other memory-related regions (19, 80). Reduced masticatory stimulation diminishes trigeminal afferent input to the CNS, which is hypothesized to attenuate neurotrophic signaling cascades (detailed in Section 4) and may accelerate neurodegeneration (29, 32). Occlusal decline also impairs the elderly’s ability to ingest hard, nutrient-rich foods, such as nuts and meat, causing deficiencies in vitamins and proteins, further exacerbating brain function deterioration (44). Crucially, it must be explicitly acknowledged that tooth loss is frequently a direct consequence of severe periodontal disease; because periodontitis itself is strongly associated with systemic inflammation and adverse neurological outcomes, its potential confounding and synergistic role deserves greater attention. Tooth loss is often accompanied by periodontitis, and lipopolysaccharides from periodontal pathogens may trigger systemic inflammation, promote β-amyloid deposition, and accelerate AD progression (19). Consequently, some researchers propose using the number of remaining teeth as a marker for dementia progression (81).

### 3.3 Occlusion and other brain function-related disorders

#### 3.3.1 Tinnitus and occlusion

Tinnitus is the perception of sound in the absence of external stimuli, with a prevalence of 10–15% in the general population (82). Studies indicate that tinnitus is associated with altered connectivity in specific brain regions (81). Occlusal interference and malocclusion can cause tinnitus. Shifted jaw positions make chewing muscles adapt and become overly tense. As a result, patients suffer from muscle pain, poor jaw movement, speaking problems, and tinnitus (83). TMD is also clearly associated with tinnitus; a systematic review found that 57.5% of TMD patients have concurrent tinnitus, with higher prevalence in painful TMD compared to non-painful TMD, and greater psychosocial distress in TMD patients with tinnitus (84). Clinical studies show that the prevalence of tinnitus in patients with masseter pain or temporomandibular osteoarthritis reaches 32.8 and 33.7%, respectively (85). Anatomically, TMD and tinnitus are connected via ligaments linking the temporomandibular joint (TMJ) and middle ear, and occlusal disturbance may affect middle ear function through these structures (84); overlapping brain pathways may further mediate these effects (86). Neurophysiologically, occlusal disturbance generates somatosensory signals that interact with auditory pathways in the brainstem, potentially altering central auditory neuron excitability. Furthermore, functional MRI studies demonstrate that somatosensory modulation via oral-facial movements or mandibular repositioning alters neural activity within the auditory cortex, such as the superior temporal gyrus, as well as limbic and salience regions, highlighting a cross-modal neuroplastic link between trigeminal/somatosensory inputs and auditory processing that serves as a potential neuroimaging marker for somatosensory tinnitus (87, 88). The central neural pathways and molecular mechanisms linking occlusal dysfunction to tinnitus remain unclear and warrant further investigation (83).

#### 3.3.2 Occlusal disturbance and cardiac dysfunction

The nervous system is tightly linked to cardiac function, with the autonomic nervous system, regulated by cortical and hypothalamic inputs, crucial for maintaining normal heart rhythm and performance (89, 90). In rodent models, occlusal disturbance elevates serum corticosterone approximately 3.6-fold in mice and increases the low-to-high frequency ratio of sympathetic activity nearly fourfold, enhancing susceptibility to atrial fibrillation and promoting atrial remodeling, including fibrosis, apoptosis, and oxidative stress (91, 92). In human studies, sleep bruxism also increases sympathetic activity, affecting heart rate variability, inflammation, oxidative stress, endothelial remodeling, and hormonal regulation, contributing to cardiovascular disease (93). Occlusal dysfunction is hypothesized to affect cardiac function and structure via neural dysregulation, endocrine imbalance, and inflammation/oxidative stress, suggesting that occlusal rehabilitation may provide a novel avenue for cardiovascular disease prevention and treatment. However, current human evidence is largely correlational, and robust causal directionality remains to be established. While direct clinical evidence remains limited, neuroimaging of the brain–heart axis suggests that central autonomic hubs, such as the insula and anterior cingulate cortex, may bridge trigeminal occlusal inputs and cardiac regulation (90), pointing to potential shared neuroimaging marker alterations and molecular cross-talk that warrant future multimodal imaging investigations.

#### 3.3.3 Occlusion and frailty

Frailty is a prevalent geriatric syndrome characterized by heightened vulnerability to external stressors (94). Fatigue and frailty are associated with dysfunction in multiple brain regions, including the PFC (executive function), insula (interoception), and basal ganglia (motor control and motivation) (95). Several studies have linked changes in dentition, occlusal force, and masseter muscle volume to frailty (96–99), though underlying mechanisms remain to be elucidated. Interventions combining oral and physical exercise improve both oral and physical functions in the elderly (100), indicating that occlusal training may be a key preventive strategy for frailty.

#### 3.3.4 Occlusion and sleep

Sleep is a complex, reversible neurobiological state influenced by biological, behavioral, environmental, and socioeconomic factors (101, 102). Occlusal status plays a significant role (103). Obstructive sleep apnea-hypopnea syndrome (OSAHS) is the most common sleep-related breathing disorder (104) and is closely associated with malocclusion. Patients often present with Class II malocclusion characterized by mandibular retrognathia, sometimes with craniofacial anomalies and narrow dental arches (105). Retrognathic Class II patients have reduced upper airway volume, aggravating obstruction (106), indicating a relationship between occlusal state and OSAHS severity (107). OSAHS Patients exhibit structural abnormalities in multiple brain regions, such as atrophy in the right brainstem, left dorsolateral superior frontal gyrus, and left superior temporal gyrus, as well as increased gray matter volume in the right inferior parietal lobule. Concurrently, they show alterations in functional connectivity, exemplified by enhanced functional connectivity between the left dorsolateral superior frontal gyrus and the right postcentral gyrus (108–110). Oral appliances, such as mandibular advancement devices (MADs), reposition the mandible, increase upper airway space, and effectively reduce apnea-hypopnea index (AHI) and daytime sleepiness in mild-to-moderate OSAHS (111, 112). MAD therapy also reduces neuronal apoptosis and increases acetylcholinesterase activity in the frontal cortex (113). However, further research with larger samples and longer follow-ups is needed to clarify the mechanisms and efficacy of MAD therapy on brain structure and function.

Occlusal disturbance may also contribute to sleep bruxism (SB), via activation of the trigeminal-brainstem pathway and disruption of serotonergic and dopaminergic systems (114–116). Peripheral sensory feedback from occlusal interference enhances rhythmic masticatory activity during sleep, exacerbating bruxism. Use of occlusal splints or mandibular advancement devices improves occlusion and sleep quality in SB patients (117). Overall, OSAHS and SB illustrate how occlusal dysfunction can affect specific brain regions and sleep physiology, and that correcting occlusion can indirectly protect brain structure and function.

#### 3.3.5 Occlusion and chronic orofacial pain

Persistent idiopathic facial pain is characterized by diffuse, continuous pain of unknown etiology, with risk factors including psychological distress, maladaptation, female sex, and adverse childhood experiences, whereas optimism may reduce risk (118). Occlusal abnormalities are considered key contributors. Occlusal dysfunction and occlusal interference overload masticatory muscles and damage the TMJ, triggering peripheral-to-central pathological cascades (119). Peripherally, tissue injury releases cytokines and ATP, activating ion channels in trigeminal ganglion neurons, leading to peripheral sensitization manifested as allodynia and hyperalgesia (120). This process is closely related to post-injury sensitization of pain fibers, neural plasticity in adjacent afferents (121), and over time, central sensitization involving glial activation in the dorsal horn, enhanced thalamic excitability, and cortical plasticity, resulting in lowered pain thresholds and pain spread (120, 122, 123). Neuroimaging confirms sustained activation of the ACC and anterior insula (AIns) in orofacial pain, with occlusal dysfunction enhancing synaptic transmission and long-term potentiation in the ACC, while chewing and pain co-activation involves coordinated activity of the left AIns, left MI, and right SI. The AIns acts as a hub for pain integration and salience processing, playing a central role in the link between occlusion-related pain and masticatory dysfunction (124).

## 4 Potential mechanisms linking occlusion and brain function

### 4.1 Peripheral sensory input

The trigeminal system forms an anatomical bridge connecting occlusion and brain function. Mechanoreceptors in the periodontal ligament and dental pulp transmit occlusal stimuli via the trigeminal ganglion to the pontine spinal trigeminal nucleus, which then projects through the ventral posteromedial thalamus to primary and secondary somatosensory cortices. The corticobulbar tract regulates masticatory muscles via the trigeminal motor nucleus, establishing a closed-loop feedback system (3, 125). This pathway underlies the biological basis of occlusal perception and mediates how oral sensory input is integrated with central neural activity. Notably, trigeminal central projections extend beyond traditional sensorimotor areas, connecting with the insula (interoceptive integration), ACC (emotional regulation), and basal ganglia (motor program control), providing an anatomical substrate for the hypothesized occlusion-related effects on emotion and cognition.

### 4.2 Central neural network regulation

Neuroimaging investigations demonstrate that tooth loss and occlusal alterations correlate with altered resting-state functional connectivity between the motor cortex and cerebellum, as well as disrupted functional connectivity within cognitive-related cortical networks, such as the PFC-centered default mode network (7, 24).

### 4.3 Molecular and cellular mechanisms

#### 4.3.1 Neurotransmitter regulation

Preclinical findings suggest that dysregulated serotonin and dopamine levels in the hippocampus and PFC are associated with compromised neuroplasticity and emotional dysregulation, potentially mediating the cognitive decline and affective disturbances associated with occlusal dysfunction (34, 66).

#### 4.3.2 Neurotrophic factors and signaling

Mastication is linked to the upregulation of BDNF, which may activate TrkB receptors to engage downstream Akt/ERK signaling pathways, thereby supporting hippocampal neuronal proliferation, synaptogenesis, and dendritic plasticity (56, 58, 126). Conversely, in animal models, reduced masticatory activity has been associated with decreased trigeminal neuron density, an observed increase in intercellular spacing by 42%, and altered neuron–glia unit integrity, which is hypothesized to impair sensory signal transduction (35).

#### 4.3.3 Inflammation and immune modulation

In preclinical paradigms, occlusal disturbance acts as an experimental mechanical and somatic stressor (65, 66, 72). It has been shown to induce neurogenic inflammation in the spinal trigeminal nucleus through trigeminal sensory afferents (3, 120, 122), and is linked to overactivation of the HPA axis to elevate systemic corticosterone levels (65, 72, 91). These pathways together are associated with the activation of central microglia and astrocytes (29, 122, 123). This non-infectious neuroinflammatory cascade is hypothesized to facilitate the local release of pro-inflammatory cytokines including IL-1β and TNF-α into key brain regions such as the hippocampus and cortex, potentially contributing to downstream neurodegenerative changes (29, 124). Notably, the above mechanistic evidence is mostly derived from animal models and preclinical studies, and robust causal validation in human cohorts remains limited. Furthermore, when occlusal trauma is combined with periodontitis, this neuroinflammatory response may be markedly exacerbated (19, 127). Biomechanical overload can accelerate periodontal tissue breakdown, which is hypothesized to facilitate the systemic and neural dissemination of circulating pro-inflammatory mediators and periodontal pathogens such as *P. gingivalis* LPS, thereby potentially exacerbating neurodegenerative progression (127).

## 5 Controversies, methodological limitations, and future perspectives

Dental occlusion and brain function exhibit complex, bidirectional relationships. From anatomical pathways to neurotransmitter and neurotrophic regulation, evidence consistently supports the interdependence of occlusal state and brain function. However, critical methodological limitations and controversies persist in current literature. Many published studies present predominantly descriptive observational summaries without rigorous quality appraisal or critical evaluation of evidence strength. In human clinical cohorts, investigations frequently fail to adjust adequately for essential confounding variables, including periodontitis, systemic inflammation, socioeconomic status, and dietary shifts. Furthermore, significant translational gaps remain, as direct extrapolation from acute, high-stress animal molar extraction models to gradual, age-related human tooth loss requires highly cautious interpretation.

**Figure 1:**
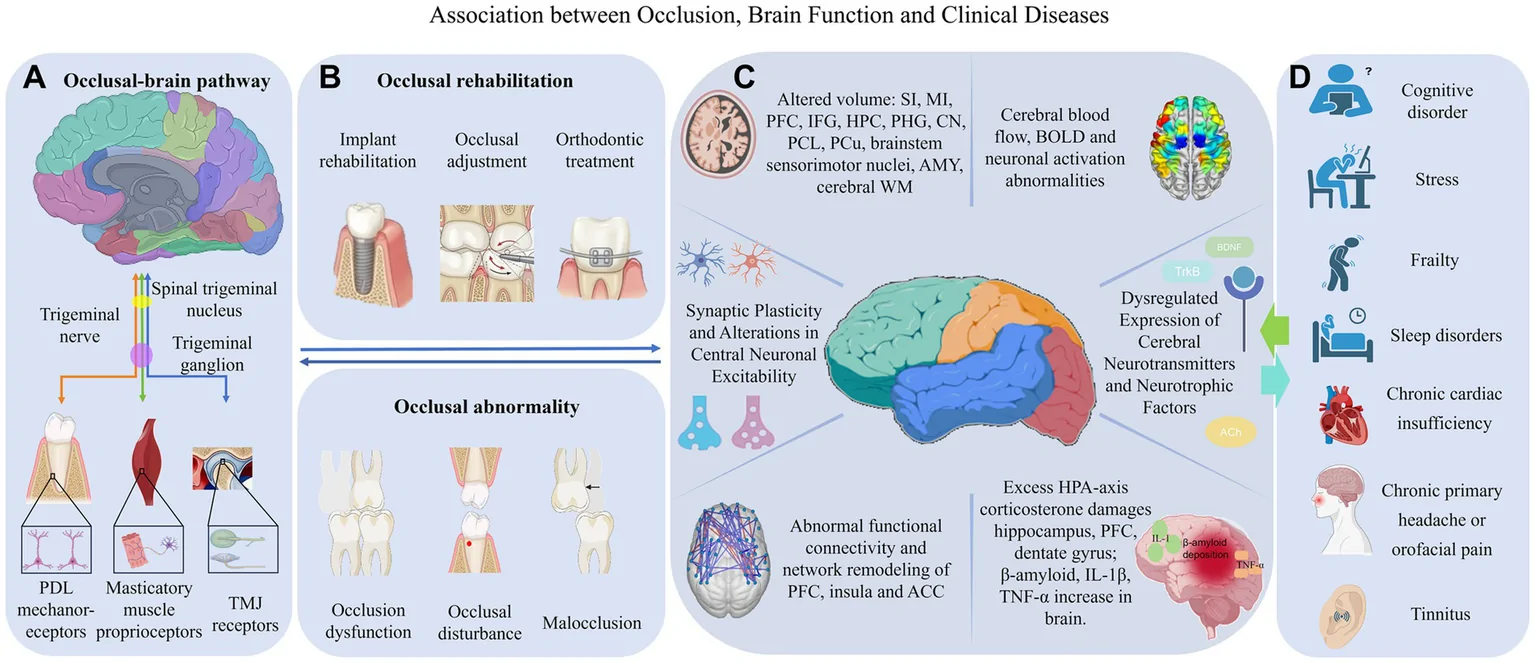
Conceptual framework of bidirectional interactions among dental occlusion, brain alterations, and clinical disorders. **(A)** Occlusal–brain pathway: Trigeminal transmission of sensory afferents from periodontal, muscular, and TMJ mechanoreceptors to the spinal trigeminal nucleus and cortex. **(B)** Occlusal states & rehabilitation: Therapeutic occlusal reconstruction (implants, adjustments, orthodontics) and pathological disruptions (dysfunction, disturbance, malocclusion). **(C)** Central adaptations & molecular cascades: Structural (regional gray/white matter volume) and functional alterations (CBF, BOLD, synaptic plasticity, PFC/insula/ACC network remodeling); dysregulated BDNF/TrkB and ACh signaling; HPA axis overactivation (excess corticosterone), neuroinflammation (IL-1β, TNF-α), and β-amyloid deposition. **(D)** Clinical comorbidities: Associated systemic and neuropsychiatric conditions, including cognitive impairment, stress, frailty, sleep disorders, cardiac insufficiency, chronic orofacial pain, and tinnitus. PDL, periodontal ligament; TMJ, temporomandibular joint; ACC, anterior cingulate cortex; AMY, amygdala; ACh, acetylcholine; BDNF, brain-derived neurotrophic factor; BOLD, blood-oxygen-level-dependent; CN, caudate nucleus; HPC, hippocampus; HPA-axis, hypothalamic–pituitary–adrenal axis; IFG, inferior frontal gyrus; IL-1β, interleukin-1β; MI, primary motor cortex; PCL, paracentral lobule; PCu, precuneus; PFC, prefrontal cortex; PHG, parahippocampus; SI, primary somatosensory cortex; TNF-α, tumor necrosis factor-α; TrkB, tropomyosin-related kinase B; WM, white matter.

To address these challenges, future research should capitalize on multimodal neuroimaging techniques, such as combining fMRI with fNIRS, to dissect the dynamic functional reorganization and neural information flow of occlusion-related brain networks across diverse occlusal states. Researchers urgently need long-term studies and standard trial tests. These projects will help confirm direct cause-and-effect links. They will test if bite treatments, like dental implants or braces, can reduce memory loss, stress, and related body diseases. Ultimately, interdisciplinary collaboration integrating dentistry, neuroscience, psychology, and rehabilitation medicine will be pivotal to unraveling the complex mechanics of the occlusion-brain axis and advancing holistic human health (Figure 1).

## Glossary

BDNF: Brain derived neurotrophic factor
SI: Primary somatosensory cortex
MI: Primary motor cortex
BOLD: Blood oxygen level dependent
SMA: Supplementary motor area
PFC: Prefrontal cortex
ACC: Anterior cingulate cortex
fNIRS: Functional near-infrared spectroscopy
HPA: Hypothalamic–pituitary–adrenal
5-HT: 5-Hydroxytryptamine
TMD: Temporomandibular disorders
fMRI: Functional magnetic resonance imaging
AD: Alzheimer’s disease
TMJ: Temporomandibular joint
OSAHS: Obstructive sleep apnea-hypopnea syndrome
MADs/MAD: Mandibular advancement devices
SB: Sleep bruxism
AIns: Anterior insula
CNS: Central nervous system
RCT: Randomized controlled trial
